# O032: Patient safety improvement in 14 african hospitals through partnerships: learning, doing and catalysing change

**DOI:** 10.1186/2047-2994-2-S1-O32

**Published:** 2013-06-20

**Authors:** SB Syed, J Storr, JD Hightower, R Gooden, S Bagheri Nejad, E Kelley

**Affiliations:** 1 WHO Patient Safety, World Health Organization, Geneva, Switzerland; 2 African Partnerships for Patient Safety, World Health Organization, London, UK; 3 African Partnerships for Patient Safety, World Health Organization, Harare, Zimbabwe

## Introduction

The 58th WHO African Regional Committee in 2008 called for action in 12 patient safety action areas, including infection prevention and control (IPC). Hospital-to hospital partnerships is a mechanism utilized by the WHO African Partnerships for Patient Safety to respond to this call, linking policy to local action in 14 African and 3 European countries.

## Objectives

Define how a partnership based approach can be utilized to catalyze patient safety improvement in African hospitals.

## Methods

Programme review of WHO African Partnerships for Patient Safety to capture key transferable lessons. The focus of the examination was on key parameters of partnership functioning to improve patient safety.

## Results

Seven key parameters of partnership functioning were identified. First, co-developing a partnership definition is critical to a strong balanced and effective partnership involving hospital teams. Second, systematic step-wise improvement necessitates a patient safety situational analysis that can be periodically repeated by the hospital team to track progress. Third, structural determinants of patient safety, for example availability of alcohol based hand rub, needs to be considered alongside training. Fourth, north-south partnership mechanisms need to be supported through strong south-south linkages to build African capacity. Fifth, IPC and specifically hand hygiene can be a tangible entry point for action on patient safety in African hospitals. Sixth, partnership improvement activities need to be aligned with national quality improvement frameworks to have a catalytic effect in national systems. Finally, recognition of culture and context are critical considerations in an effective and mutually beneficial partnership.

## Conclusion

Early programme implementation has focused on learning and doing at the same time. The urgency of patient safety in African hospitals negates the possibility of lengthy project design phase. Multiple co-developed resources and tools are now available that can be utilized by any hospital-to-hospital partnership focused on patient safety. A technical platform now allows hospital partnerships to join a global community to learn, do and catalyse change, together.

## Disclosure of interest

None declared.

